# Editor's Note on ‘MicroRNA-296 is enriched in cancer cells and downregulates p21WAF1 mRNA expression via interaction with its 3′ untranslated region’

**DOI:** 10.1093/nar/gkaf029

**Published:** 2025-01-24

This an editor's note regarding: A-rum Yoon, Ran Gao, Zeenia Kaul, Il-Kyu Choi, Jihoon Ryu, Jane R. Noble, Yoshio Kato, Soichiro Saito, Takashi Hirano, Tetsuro Ishii, Roger R. Reddel, Chae-Ok Yun, Sunil C. Kaul, Renu Wadhwa, MicroRNA-296 is enriched in cancer cells and downregulates p21WAF1 mRNA expression via interaction with its 3′ untranslated region, Nucleic Acids Research, Volume 39, Issue 18, 1 October 2011, Pages 8078–8091, https://doi.org/10.1093/nar/gkr492

In November 2024, the Editors were informed of two concerns, also posted on PubPeer (https://pubpeer.com/publications/B5D5AA520B84BFB0F4C8C49243FE8B), regarding image alteration and duplication in Figures 5A and 5C, as outlined below.

**Concern 1**. A portion of a gel strip appears to have been used in both Figure 5A (U-2OS miR-296) and a panel within Figure 5C (U-2OS p21WAF1) after rotation, cropping and aspect ratio changes.

The authors could not locate the original data for Figure 5A (U-2OS miR-296) but provided similar data from a repeat experiment performed at the time of revision, available on page 1 and 2 of the accompanying supplementary data.

The original data for Figure 5C (U-2OS p21WAF1) was supplied and is shown on page 3 of the supplementary data.

**Concern 2**. The same GAPDH controls seem to have been used for different samples in Figure 5C.

The authors could not find the original data for the MCF 7 and U-2 OS experiments but provided the original data for the JFCF 6-6B and JFCF 6-3C experiments, shown on page 4 of the supplementary data.

The original data was partially lost during the earthquake and tsunami of March 2011 and the subsequent lab relocation. The Editors’ analysis of the similarities between Figures 5A and 5C after rotation and stretching (Concern 1) are inconclusive. However, the analysis of the GAPDH blots in Figure 5C (Concern 2) supports the idea that these were duplicated during image assembly.

In conclusion, while these issues may not impact the results or conclusion of the study, in the absence of some original data, the Editors advise readers to examine Figure 5 with care.

**Julian E. Sale and Barry L. Stoddard**

Senior Executive Editors

## Supplementary Material

gkaf029_Supplemental_File

