# Supplementary material for: Editor's Note on ‘MicroRNA-296 is enriched in cancer cells and downregulates p21WAF1 mRNA expression via interaction with its 3′ untranslated region’
Source: Nucleic Acids Res. 2025 Jan 24;53(3):gkaf029. doi: 10.1093/nar/gkaf029 (PMC11760941; doi:10.1093/nar/gkaf029)
Supplement: gkaf029_Supplemental_File [file gkaf029_supplemental_file.pdf]

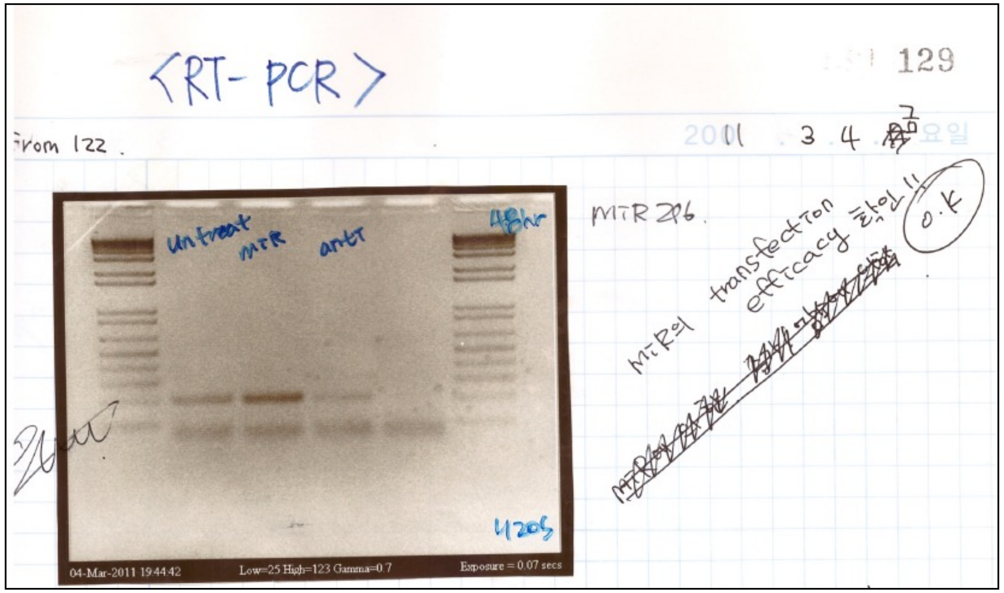

Original scan

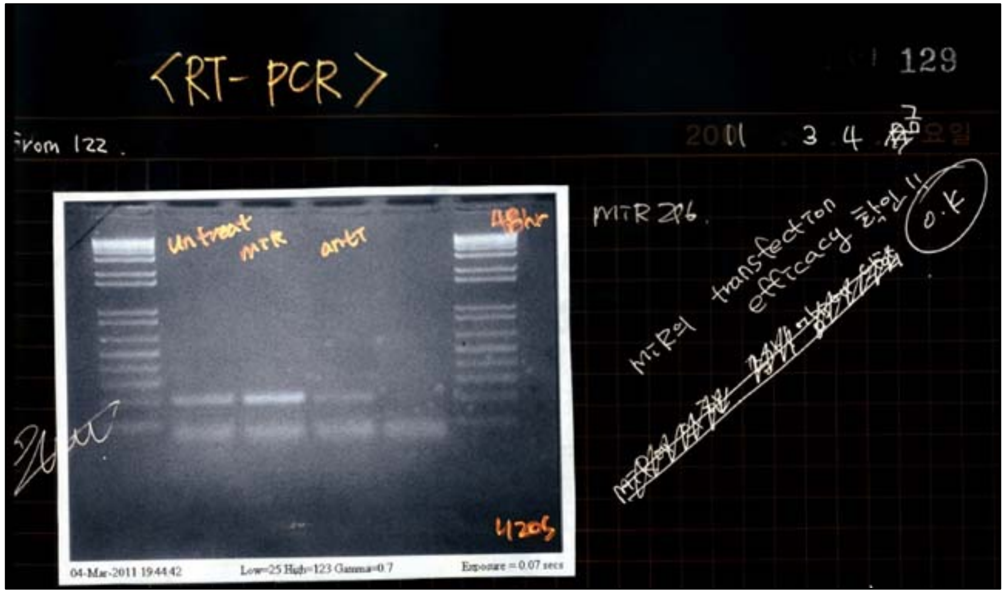

Converted version

Legend- Scan of the lab note recorded on 2011/03/04 (during the revision of the manuscript) showing the data consistent with those shown in Fig. 5A.

Please note that during those days in our lab, we could not obtain digital images directly from the transilluminator, gel images were printed out and pasted directly into lab notebooks as raw data.

We scanned the relevant pages (#129; shown above and #121 shown in the next page) from lab note #44.

From120

121

"RT-PCR" 진행과정.

2011 . 2 . 19 . 토 요일

① RNA 정량 과 CDNA 합성.

	conc.	1μg	olig dT	dNTP	DW
untreated	191.6	5.2	1	4	2.8
miR 296 (24 hr)	224.6	4.5	1	4	3.5
Anti-miR	211.7	4.7	1	4	3.3
miR 296 + Anti	188.3	5.3	1	4	2.7
miR 296 (12 hr)	401.4	2.5	1	4	5.5
miR 296 (36 hr)	235.9	4.2	1	4	3.8
miR 296 (48 hr)	295	3.4	1	4	4.6

실험조건

RNA 1ug . 8 ul.
oligodT 1 ul.
dNTP mix 4 ul
13 ul

65°C, 5 min →

ul.
RNase Out . 1
RT 2
5x Bfr 4
20 ul

→ 37°C 1 min . → CDNA 합성 완료!!

go to 128

2011. 2. 22. (화)

<PCR>

PCR for → p21
→ miR

x 8.5

10x PCR Bfr 5
2.5mM dNTP 4
primer 1
Taq 1
D.W 37
50

95°C, 1 min
60°C 1 min
72°C 1 min.
28 cycle!!

From go to 123

<RT-PCR>

129

2011 . 3 . 4 금 요일

From 122.

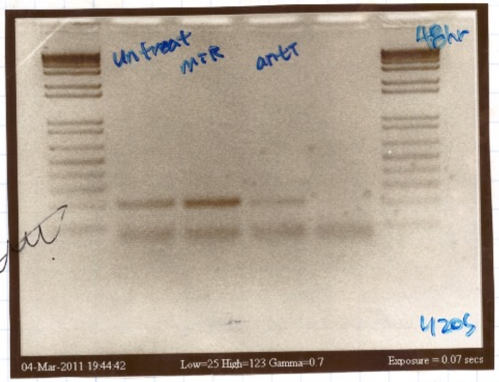

miR 296.
miR의 transfection efficacy 확인!! O.K

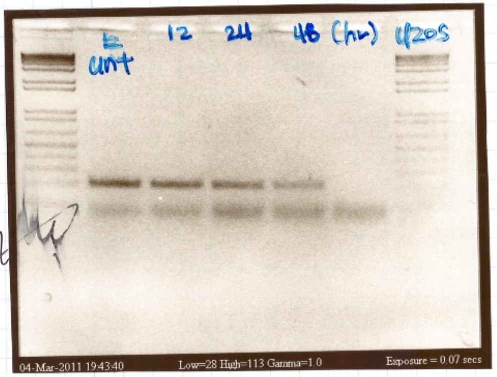

p21.
half 나오는 RT-PCR로 확인하자!!
go to 128.

Legend- Scan of the lab note #44 relevant pages (# 121 and #129) for data shown in slide1. Please note that the lower panel is time-dependent response which is not included in this manuscript.

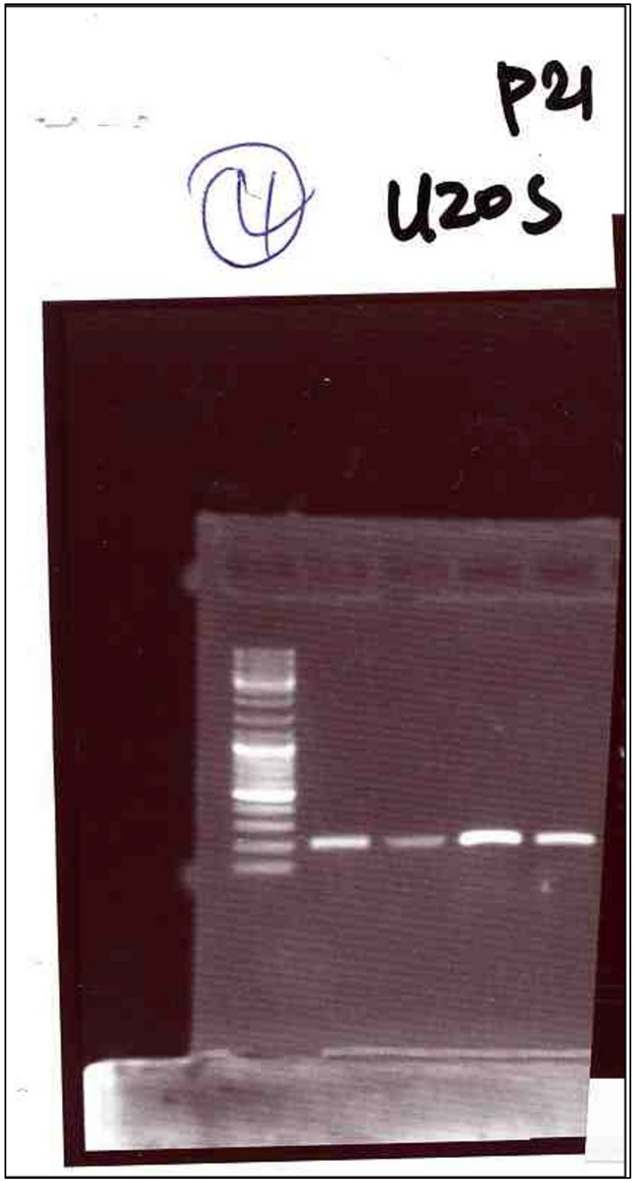

Legend- Scan of the lab note from Year 2009 (file name 090212-1120-U2OS-p21 showing raw data of Figure 5C (U-2OS p21^{WAF1}).

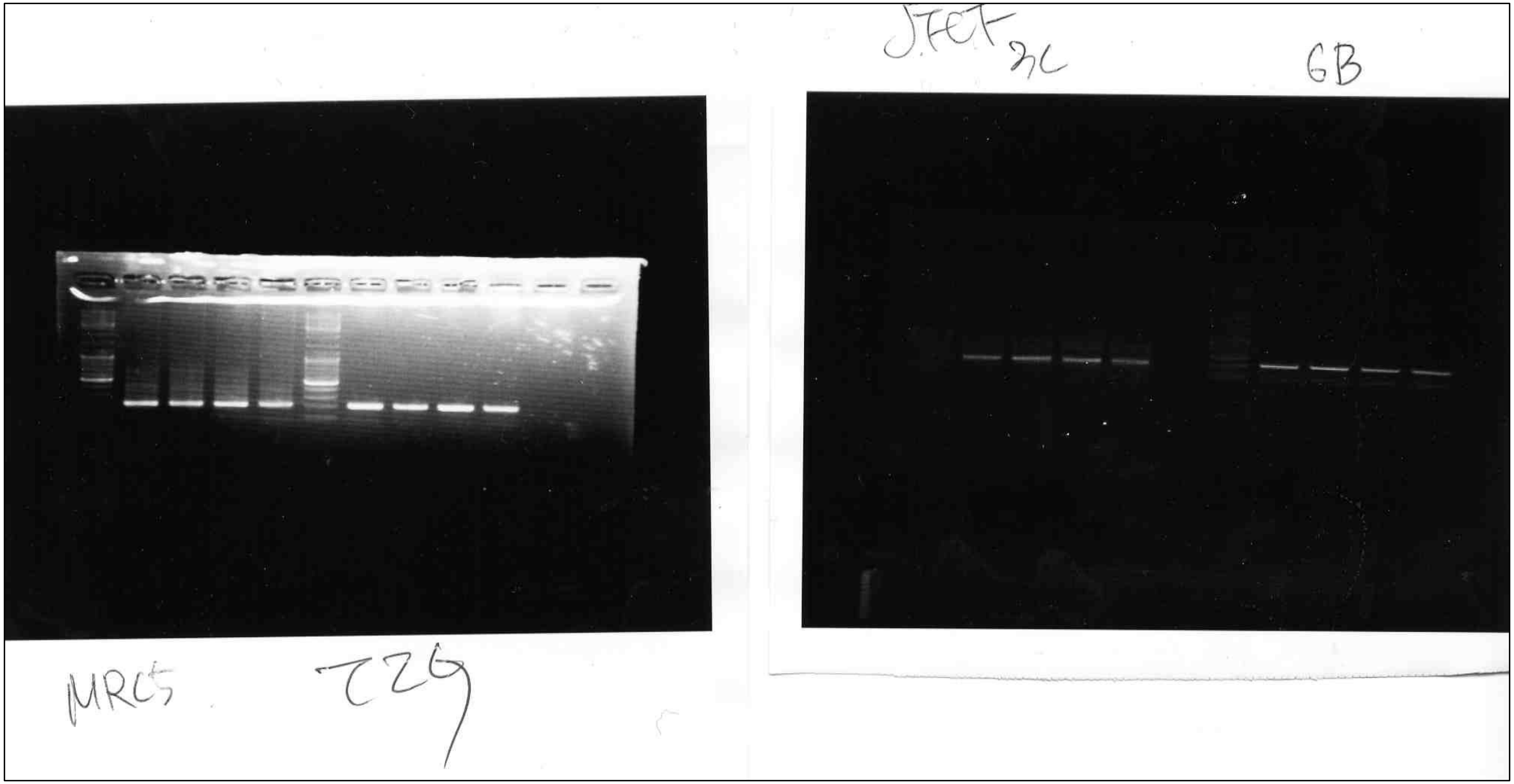

Legend- Scan of the lab note from year 2009 (File name GAPDH190_Fig.5C) showing raw data of GAPDH shown in Figure 5C.
